# The Safety, Technological, Nutritional, and Sensory Challenges Associated With Lacto-Fermentation of Meat and Meat Products by Using Pure Lactic Acid Bacteria Strains and Plant-Lactic Acid Bacteria Bioproducts

**DOI:** 10.3389/fmicb.2019.01036

**Published:** 2019-05-08

**Authors:** Elena Bartkiene, Vadims Bartkevics, Erika Mozuriene, Vita Lele, Daiva Zadeike, Grazina Juodeikiene

**Affiliations:** ^1^Department of Food Safety and Quality, Institute of Animal Rearing Technologies, Lithuanian University of Health Sciences, Kaunas, Lithuania; ^2^Laboratory of Food and Environmental Investigations, Institute of Food Safety, Animal Health and Environment “BIOR”, Riga, Latvia; ^3^Department of Chemistry, University of Latvia, Riga, Latvia; ^4^Department of Food Science and Technology, Kaunas University of Technology, Kaunas, Lithuania

**Keywords:** lacto-fermentation, meat and meat products, plant-lactic acid bacteria bioproducts, safety, biogenic amines

## Introduction

Lactic acid bacteria (LAB) are the most popular microbial cultures used in the preparation of fermented foods (Bintsis, 2018). Due to their wide range of antimicrobial activity, LAB have been shown to improve safety, nutritional and sensory characteristics, control fermentation by microflora and speed maturation, as well as increase the shelf life of products (Des et al., 2018). Recently, as a new approach for the application of technological starters, great interest has been concentrated on their biodegradation and/or absorption properties of non-desirable chemical compounds, and it was reported that LAB can reduce polycyclic aromatic hydrocarbons (PAHs) and heterocyclic aromatic amines (Bartkiene et al., 2017; Chiocchetti et al., 2018). However, fermented meat and meat products can be a source of biogenic amines (BAs), and relatively high concentrations of these compounds were reported in fermented sausages (EFSA, 2011; Özogul and Hamed, 2018), as well as in other foods (Capozzi et al., 2012; Lee et al., 2016; Li et al., 2018). Low concentrations of BAs are not toxic; however, putrescine and cadaverine can form carcinogenic nitrosamines, especially in meat products, where nitrite is used as a technological compound, also polyamines and diamines can form stable N-nitroso compounds (Eliassen et al., 2002). Therefore, the search for solutions to reduce undesirable microorganisms, as well as to decrease BAs and PAHs and control their levels in foods is very important. Also, attention has been paid to the meat products, with functional, as well as health promoting compounds, technologies development. For this reason, health enhancing ingredients such as plant based antioxidants, dietary fibers, and savory plants are rapidly increasing worldwide similar to natural bio-preservatives and value enriching compounds. Finally, it is very important to evaluate the risk of technological processes and to analyse the safety parameters of the final products, because separate ingredients can be general recognized as safe; however, during the technological processes various changes, desirable and non-desirable, can be initiated.

## Lactic Acid Bacteria for Biodegradation of PAHs and BAs in Cold Smoked Pork Sausages

The influence of a treatment with *pediococcus* and *lactobacillus* strains (*Pediococcus acidilactici* KTU05-7, *Pediococcus pentosaceus* KTU05-9 and *Lactobacillus sakei* KTU05-6), previously cultivated in an alternative to the expensive de Man, Rogosa, Sharpe (MRS) broth substrate—in juice of potato tubers, on the formation of PAHs and BAs in cold smoked pork meat sausages was investigated (Bartkiene et al., 2017). Due to their low cost and good nutritional quality, potatoes are very popular vegetables, and play an important role in human nutrition (Kim et al., 2012), and their byproducts (juice) can be adapted as a substrate that is rich in nutrients for technological cultivation of microorganisms. In the experiment under discussion, the LAB starters used inhibited the growth of *Pseudomonas aeruginosa, Escherichia coli, Staphylococcus aureus, Salmonella enterica serovar typhimurium, Bacillus cereus, Listeria monocytogenes, Yersinia enterocolitica*, and *Y. pseudotuberculosis*. The treatment of sausage surfaces with KTU05-7, KTU05-9, and KTU05-6 before smoking decreased the content of cadaverine (CAD) and spermidine (SPRMD), whereas the treatment after smoking process reduced the content of putrescine (PUT) or totally eliminated it (by using KTU05-9) from the sausages (from the both: outer and center layers). The application of LAB for sausages treatment (before or after smoking) significantly decreased chrysene and benzo[a]pyrene concentration in this type of meat products. The interest of the biodegradation of toxic compounds by using microorganisms is growing. It was published about the possible LAB properties to directly degrade PAHs and heterocyclic aromatic amines (Abou-Baker et al., 2012; Abou-Arab et al., 2015). Also, the use of BLIS producing LAB can control of the fermentation microflora etc. technological parameters of the process, and indirectly reduce BAs formation in foods, which, in many cases, depends on the activity of the spoilage bacteria, as well as technological starters, which decarboxylase activity is high. Finally, it was confirmed that the potato juice is suitable substrate for KTU05-7, KTU05-9, and KTU05-6 cultivation, and the obtained fermented bioproducts can be used for reducing microbial contamination, PAHs, and BAs in cold smoked pork sausages.

## Marinade Based on With LAB Fermented Potato Juice for Pork Meat Treatment

Marination is a useful technology for meat treatment to increase water-holding capacity (WHC), tenderness and flavor (Mozuriene et al., 2016). The main idea of a previous study by Mozuriene and co-workers was to develop a natural marinade, based on selected LAB strains (KTU05-7, KTU05-9, and KTU05-6) cultivated in potato juice, for pork meat treatment and to evaluate the WHC, pH, cooking loss, color, tenderness, and sensory characteristics, as well as BAs content in the marinated pork meat. Significant differences were demonstrated between the quality parameters of the marinated and non-treated pork meat samples. Pork meat marination (24 h) with developed potato juice—LAB marinade, lowered the WHC of meat, thus increasing cooking loss, as compared to the non-marinated samples. Also, marination reduced the lightness and the yellowness; however, increased the redness and improved sensory characteristics of pork meat. Phenylethylamine, putrescine, and cadaverine were predominant BAs in all pork meat samples; however BA concentrations were far below the levels considered to cause a health risk. Finally, cheap natural marinades based on potato juice fermented by selected *Pediococcus* technological starters can be recommended for pork meat treatment to improve meat quality and safety.

## Lacto-fermented Tomato Powder for Ready-to-cook Minced Meat Products Enrichment

Increasing consumer demand for high quality and nutritious meat products has led to development of new meat treatment technologies and new natural and safe ingredients for meat products. For this reason, an aim of a previous study by Bartkiene and co-workers was to develop bioproducts based on lacto-fermented tomato powder, to preserve meat products from spoilage microorganisms and to enrich meat products with desirable antioxidants and to ensure higher redness (Bartkiene et al., 2015a). Tomatoes are a rich source of lycopene, which is a natural carotenoid and possesses various health benefits. For this reason, the influence of fermentation on the quality of tomato powder (TP) was evaluated, and the effect of adding fermented TP to ready-to-cook minced pork meat products (RCMP) to improve their quality characteristics was analyzed. The use of the above mentioned LAB starters increased the β-carotene and lycopene concentrations in TP, on average by 45%, compared with the non-fermented TP samples. Lycopene and β-carotene contents in the RCMP were proportional to the TP added (10 and 30%). Fermentation with KTU05-6 TP at a level of 10% can be recommended for RCMPs preparation as a coloring agent and a source of lycopene, as the use of KTU05-9 and KTU05-6 technological starters increased the carotenoid content in fermented TP, which is a beneficial additive that improves the color and nutritional value of RCMPs. This can be a new approach for RCMPs enrichment by using fermentation with selected LAB TP as a source of lycopene and β-carotene, and more research is needed to explain the mechanism of carotenoid increases during fermentation of TP.

## Lacto-fermented *Helianthus tuberosus* L. Tubers for Enrichment of Ready-to-cook Minced Pork Meat Products

*Helianthus tuberosus* L. (HT) is a plant consisting of the various compounds: carbohydrates (the main inulin), proteins, lipids, and macro- and microelements as minor constituents of potent physiological activity (Paseephol et al., 2007), and fermented HT, by using bacteriocin-like inhibitory substance (BLIS) producing LAB starters, can be a good source for functional, as well as nutritional value improvement of meat. For this reason, the main idea was to evaluate the influence of fermentation of HT tubers with KTU05-7, KTU05-9, and KTU05-6 on the characteristics of RCMP (Stimbirys et al., 2015). It was established that the fermented HT additives reduced pH of the RCMP and decreased WHC from 2.01 to 2.93%. Concentrations of BAs in RCMP with fermented HT additives were significantly lower compared with control samples, and were far below levels associated with a health risk. The count of undesirable microorganisms in meat samples was significantly reduced in the presence of LAB-fermented HT bioproducts. Also, the composition of volatile compounds (VC) of RCMP without HT additive and with LAB-HT additives was analyzed. The results of sensory analysis of RCMP enriched with LAB-HT additives revealed that pleasant and acceptable flavors for consumers might be explainable by LAB-HT additives leading to accumulation of VC such as undecane ethylbenzene, decane, toluene, and 2-methylundecane. While, N-morpholinomethyl-isopropyl-sulfide, 6-undecilamine, and N,N-dimethyl-1-pentadecanamine were not detected in RCMP with LAB-HT additives. Finally, the addition of LAB-HT bioproducts to RCMP increased the formation of VC and improved the sensory characteristics of RCMP samples. According to these results, 5% of the KTU05-7 starter fermented HT additive can be recommended for RCMP preparation to prevent microbiological spoilage and increase the overall acceptability (VC concentration) and shelf-life.

## Lacto-fermented *Satureja montana* L. for Improving Value of Ready-to-cook Minced Pork Products

*Satureja montana* L. antimicrobial properties are associated with carvacrol, terpinen-4-ol, thymol, and linalool (Dorman and Deans, 2000; Azimi et al., 2018; Pateiro et al., 2018). As combinations of antimicrobial compounds might promote increased effectiveness of the antimicrobial products and allow reduction of the dose of each compound needed, another experiment was performed by using the *Satureja montana* L. and antimicrobial properties having LAB compositions to improve meat products safety and quality. The main idea of this study was to perform solid state fermentation (SSF) and traditional submerged fermentation (TF) with *Satureja montana* L. plants (SMP) with antimicrobial technological starters, and to evaluate the effect of fermented SMP additives on RCMP quality and safety parameters (Bartkiene et al., 2015b). It was established that the viability of LAB in SMP medium significantly depended on the type of fermentation (TF or SSF). Supplementation of RCMP with SSF SMP reduced the growth of mesophilic bacteria up to 34% during 120 h storage, while TF SMP additives had a lower effect (up to 17.4%). The highest antimicrobial activity against pathogens was shown by SSF SMP additives fermented with KTU05-7. The significantly higher overall acceptability of RCMP samples prepared with 3% of SSF SMP was established. The addition of SMP increased tenderness and WHC, as well as enriched the RCMP with ρ-cimene, γ-terpinene, and carvacrol. Both types of SMP fermented with tested technological LAB starters significantly reduced the total BAs concentration in RCMP (to 0.3 mg/kg dry weight) compared to control samples (43.96 mg/kg dry weight). Finally, the supplementation of meat products with savory plants, LAB bioproducts, can be a new approach and good alternative for RCMP processing to prevent meat decoloration, increase shelf-life, phenolic compound contents and overall acceptability.

## Conclusions

There are many possibilities for preparation of fermented meat products and the main technological starters in these processes are LAB. Usually LAB have a GRAS status and have many advantages; however, the final product should be evaluated to ensure low concentrations of BAs. Also, combinations of selected technological starters and plant based ingredients can be very promising for increasing values of meat products. In addition, by using different antimicrobial compounds it is possible to promote effectiveness of the antimicrobial properties and reduction of the dose of each compound. Finally, the formation of undesirable compounds during the manufacturing process is unavoidable, however, specific technological solutions may be encouraged to mitigate these issues and control undesirable compounds formation till non-toxic concentrations.

## Author Contributions

EB worked on the development of food technologies. VB worked on the creation of new analytic methods. EM worked on the physical chemical analysis of the food samples. VL worked on the lactic acid bacteria isolation and characterization. DZ worked on the physical chemical analysis of the food samples. GJ worked on the development of food technologies.

### Conflict of Interest Statement

The authors declare that the research was conducted in the absence of any commercial or financial relationships that could be construed as a potential conflict of interest.
